# “Where exactly do I fall?”: understanding intersectional marginalized identities through Asian Americans’ experiences

**DOI:** 10.3389/fpsyg.2024.1433156

**Published:** 2024-12-18

**Authors:** Bin Zhang, Wenqian Du, Bo Chang

**Affiliations:** ^1^School of International Education, Shandong University, Jinan, China; ^2^Teachers College, Ball State University, Munich, IN, United States

**Keywords:** intersectionality, racism, sexism, heterosexism, intersectionalism, Asian Americans, LGBTQ, internalization

## Abstract

The purpose of this study is to understand the intersection of race, gender, and sexual orientation through exploring the experiences of Asian American female sexual minority (AAFSM) college students in the U.S. Midwestern universities. We employed one of McCall’s three intersectionality methodological approaches—*intra-categorical complexity*—to guide the study. The findings indicated that the AAFSM student participants had experienced intersectional objectifications, including racial, gendered, and sexual objectification. The finding answered our research question regarding what it is like to be AAFSM students attending predominantly White campuses in Midwestern universities. Our findings also showed that the participants intersectionally internalized racism, sexism, and heterosexism as strategies to avoid being ostracized, which in turn allowed society/institution to internalize these -isms as norms. The manifestations of intersectional internalizations reported by participants included racial stereotypes, Whitenization, stereotypical gender roles, gender norms, parental influence, and institutional influence. The findings also indicated that the participants experienced intersectional blindness, which affected their understanding of Asianness, womenness, and LGBTQness. Another meaningful finding was that the racism, sexism, and heterosexism experiences of the AAFSM students were compounded and complex. The theme was best categorized as intersectional post-racism-(hetero)sexism. We discussed intersectional internalization; de-intersectional-internalization; re-intersectional-internalization; intersectional visibilities, connections, and representations; the implementation of intersectionality; and intersectionalism. The discussions, as a milestone, provided meaningful suggestions for educators, administrators, and universities to effectively create an intersectional inclusive educational environment for AAFSMs.

## 1 Introduction

*Intersectionality*, as either a theoretical framework or an analytical tool, has been utilized by researchers to gain an expansive understanding of individual’s lived experiences (Windsong, 2016), identity development (Duran and Jones, 2019), and multiple exclusions and oppressions (Collins and Bilge, 2018). Crenshaw (1989) took a new look at inequality and realized that it should not be understood along only one dimension, such as race or gender. Although not the first person to introduce the idea of intersectionality, Crenshaw is still widely regarded as a leading scholar who employed the notion of intersectionality in inequalities. Crenshaw’s (1989) analogy of traffic in an intersection, the metaphor of race and gender ambulances (2012), and the metaphor of a basement (1989) indicated that a single-axis analysis (e.g., focusing on gender or race only) does not suffice when discussing about inequality. Crenshaw (1991) critically advanced that intersectionality, as a transitional concept and methodology, should remedy how mutually exclusive categories are falsely separated for the purpose of countering the disembodiment of multiple oppressed groups at the intersection. Since then, the term *intersectionality* has been used by scholars across various avenues and has become a “buzzword” (Davis, 2008, p. 75; Nash, 2008, p. 3). Therefore, an increasing number of studies have discussed intersectionality by emphasizing two dimensions of various marginalized categories, including but not limited to, African American women (Crenshaw, 1989), Asian American gay men (Eguchi and Spieldenner, 2015; Han et al., 2014; Hom, 1994; Wooden et al., 1983), and Black gay men (Squire and Mobley, 2014).

Specifically, regarding the intersection of race and sexuality, Han et al. (2014) stated that Asian American LGBTQ people experienced “high levels of both racism in the gay community and homophobia in their ethnic communities” (p. 53). Icard (1986) also found that African American gay males struggled to develop a positive self-identity due to both homophobia in the Black community and racism in the gay community. In addition, Wooden et al. (1983) pointed out that Japanese American gays received little support and were not tolerated in their Japanese community and were viewed by White gay men as others. Regarding the intersection of race and gender, Crenshaw (1989) pointed out that African American women were marginalized by both Black and women communities. Regarding gender and sexual orientation, an interview from Hom (1994) explained how lesbians were oppressed. The interviewee was a Japanese American woman who grew up in Hawaii with her lesbian daughter. When asked about the people’s attitudes toward them, she said, “They look down on those gays and lesbians, they make fun of them…. It seems as if it is an abnormal thing … They call her a tomboy because she’s very athletic and well built” (p. 40).

However, within academia, intersectionality should not be utilized as a “buzzword” or overhyped trend. Instead, intersectionality is meant to challenge the prevailing mindsets of the dominant groups and disrupt the normative claim of dominant discourses (e.g., heteronormativity, masculinity, and Whiteness). Intersectionality should “disorient” us to “get us thinking about how ‘we’ think” (Carastathis, 2019, p. 111). Drawing on Ahmed’s (2007) concept of disorientation, intersectionality should be deployed in research to deconstruct and disorient our entrenched cognitive habits. For instance, how does one understand and make sense of normalized and exalted identities (e.g., male, heterosexuality, White people, etc.) compared to pathologized and repressed identities (e.g., female, LGBTQ, non-White)?

When applying Crenshaw’s African American women argument onto Asian American female sexual minority (AAFSM) population, one acknowledges that AAFSM might neither speak for *all* women, *all* Americans, *all* Asians, nor *all* sexual minorities due to their intersectional identities. AAFSM students’ educational experiences can be both similar to and different when compared to the experiences of White women, Asian American men, and White LGBTQ. AAFSMs sometimes share experiences of discrimination similar to those faced by White women, or those by Asian American men, or sometimes by sexual minorities.

Very limited research has documented how all three factors of race, gender, and sexuality intersectionally affect individuals such as those from AAFSM. Thus, there remains a serious lack of exploration and intervention on this population. While exploring minority experiences, it is crucial to take into account a person’s other marginalized identities because they may be “a minority within a minority” (Greene, 1997). For instance, acknowledging the experiences of LGBTQ students as sexual minorities may lead to a greater potential for exclusion in communities where the racial minority identity of LGBTQ people of color may be denied (Miville and Ferguson, 2006). Thus, it is crucial to simultaneously analyze the intersections of race, gender, and sexual orientation when exploring the experiences of AAFSM.

This research sought to contribute to the current literature by expanding variation scales from two dimensions to a more complex fashion. To this end, this study intended to draw attention toward an unmarked marginalized group—AAFSM—to explore how their personal identity, college experiences, and educational opportunities were co-constructed with race, gender, and sexual orientation. Qualitative research was conducted in several Midwestern universities to examine these aforementioned aspects. Employing a qualitative methodology and intersectionality framework, the researcher interviewed nine AAFSMs attending Midwestern colleges in the United States to gain a deeper understanding of their lived experiences.

## 2 Research design

This study seeks to better understand the educational and lived experiences of AAFSM students by exploring, describing, and explaining how they make sense of their school lives while managing their intersectional identities. To this end, we design three research questions. They are:

1. What is it like to be an Asian American female sexual minority in a predominately White Midwestern university?
2. How do race, gender, and sexual orientation intersectionally shape the lived and educational experiences of Asian American female sexual minority college students?
3. How do Asian American female sexual minority students with multiple identities navigate through previous and present schooling and what identity steers this navigation (e.g., race, gender, or sexual orientation)?

### 2.1 Intersectionality as a methodological approach

Intersectionality, as an emerging methodological tool, explores how multiple oppressed groups, for example AAFSMs, are politically, socially, and educationally relegated to the bottom of multiple social hierarchies (e.g., the gender hierarchy, sexuality hierarchy, and racial hierarchy). The purpose of using intersectionality as a methodological tool was to counter the disembodiment of AAFSMs from race, gender, and sexual orientation. McCall (2005) pointed out that intersectionality involves multiple dimensions of social life and categories. Therefore, one of the distinctive characteristics of intersectionality research is its complexity. For the sake of comprehension and manageability, McCall (2005) introduced three methodological approaches to explore the complexity of intersectionality in social life by utilizing analytical categories such as race, gender, and the like. They were *anti-categorical complexity*, *intra-categorical complexity*, and *inter-categorical complexity*. This study employed *intra-categorical* complexity and the reasons were further examined.

*Anti-categorical* complexity criticized the usage of analytical categories such as race and gender, due to their indefiniteness, fluidity, and complexity. We accepted the fluidity and complexity of individual’s analytical categories, nevertheless, we adopted analytical categories because of our focus on analyzing “the relationships of inequality among already constituted social groups…to explicate those relationships” (McCall, 2005, p. 1785). Both *inter-categorical* and *intra-categorical complexity* methodological approaches adopt analytical categories, however, the former focuses more on the multiple social groups within and across analytical categories, whereas, the latter emphasizes a single dimension of each category. For instance, researchers, who embraced the *inter-categorical complexity* may incorporate sexuality as an analytical category; then, heterosexuality and homosexuality will be compared systematically. Since the aim of this approach is to investigate the multiple groups within and across analytical categories, the other analytical categories also need to be incorporated, such as the race of Asian Americans. Next, researchers need to analyze the multiple ethnic dimensions within that racial category—say, Chinese Americans, Korean Americans, and Asian new wavers. Thus, the number of groups expands to six due to cross-classification with sexuality category (i.e., heterosexual Chinese Americans, heterosexual Korean Americans, heterosexual Asian new wavers, homosexual Chinese Americans, homosexual Korean Americans, and homosexual Asian new wavers). Our study attempted to explore the intersection of race, gender, and sexual orientation; however, if this study employed *inter-categorical complexity* to explore AAFSM college students’ experiences by analyzing across race, gender, and sexuality and within analytical categories (i.e., Asian American, Korean American, Asian new wavers, women, men, gay, lesbian, bisexual, transgendered, and questioning individuals), the *inter-categorical complexity* would expand the number of groups to 30, making the study unmanageable, incomprehensible, and complicated.

Therefore, this study limited the scope to one dimension of each category. One category could be Asian American from the racial category, women from gender, and sexual minority from sexuality, thus managing the complexity in this study.

### 2.2 Multi-cases studies and research participants’ description

This study employed multi-cases studies design, which limited the parameters of research participants to AAFSM college students and the research sites to predominantly White Midwestern universities.

Nine AAFSM students from Midwestern universities in the U.S were recruited. One participant was recruited through typical recruitment method—participant-to-researcher method. The other participants were recruited using campus-wide email and contacting university organizations, such as the Asian American Association, women’s club, and the Spectrum (LGBTQ+) Organization. Snowball sampling was also used to recruit potential participants who were not active in either the LGBTQ community, the Asian American community, or women’s community on campus, wherein research participants refer other AAFSM college students they know.

Eventually, nine AAFSM students from Midwestern universities in the U.S were recruited. Three self-identified as female bisexual Chinese American; two identified themselves as female bisexual Filipino Americans; one believed she was a gender fluid demi-homosexual Chinese American; the other three self-identified as female queer Filipino American, gender fluid bisexual Asian American, and non-binary lesbian Korean American.

### 2.3 Instruments

In-depth interviews comprising open-ended questions were appropriate for capturing detailed, holistic, and comprehensive descriptions of AAFSM college students’ educational experiences regarding race, gender, and sexual orientation from an intersectional perspective. In order to measure the effectiveness of the interview protocol, clarity of wording, convenience of the interview setting, length of interviews, and adequacy of answers, a pilot study was conducted with an Asian American gay man who had graduated from a Midwestern university. To achieve these goals, after the interview, the participant from the pilot study was asked several questions to make any needed modifications or adjustments. After the responses were collected, appropriate changes were made in the interview protocol to clarify the interview questions and improve the efficiency of the interviews. For example, to ensure the participants did not lose interest, the interview time was limited to range from 60 to 90 min.

Data were collected through one-to-one interviews of AAFSM college students. The participants were asked open-ended questions to collect narrative data. During the data collection process, the researcher paid strict attention to the *intra-categorical complexity* by asking questions about the intersection of race, gender, and sexual orientation aligning with racism, sexism, and heterosexism. Instead of asking race-related, gender-related, and sexuality-related questions separately, giving emphasis to single-axis, participants were encouraged to think about race, gender, and sexuality in line with the intersection of racial, sexual, and gendered social practices simultaneously. In addition, this research employed Crenshaw’s (1991) data collection strategy, which asked participants to think about their lived experiences on campus from structural, representational, and political aspects.

Nine AAFSM college students were interviewed until saturation of information was reached. The initial interviews were followed by at least one phone call or face-to-face follow up to check for any further clarification or explanation. Interviews were audio-recorded and transcribed carefully and precisely in order to generate data, code, cluster codes into themes, and summarize findings. All interview recordings were secured in a personal laptop with passcode access to protect the confidentiality of respondents. This laptop was locked in a secure location when not in use. All recordings will be deleted and transcripts will be shredded after 5 years.

### 2.4 Data analysis

In the data analysis stage, qualitative data analysis application was utilized—MAXQDA 2023—to work on data analysis process and Saldana’s (2015) coding types were employed to analyze data. The coding types comprised process coding, narrative coding, dramaturgical coding, causation coding, value coding, domain and taxonomic coding, emotion coding, versus coding, concept coding, initial coding, *in vivo* coding, descriptive coding, subcoding, magnitude coding, and attribute coding. The second coding cycle provided themes from these code segments.

## 3 Findings

It’s a very interesting time, cause in the United States, it’s, it’s just the minority group stepped on top of minority group on another minority group because you know I’m not White. I’m not heterosexual. And I’m not a man. And so, they just kind of get put together. And sometimes I think what kind of mean joke was this? Like who gave me all of these intersecting minority identities and then put me in a country where I’m not a majority? (Lyric, Pos. 16).

This was the response captured from a bi Asian American woman when she was asked what it was like to be an AAFSM in a predominantly White university in the U.S. Others were asked the same question, and the most frequently cited experiences disclosed by research participants might be best categorized as intersectional exclusions. This frustrated the participants, who believed that these exclusions were directly related to *intersectionalitism*, which includes intersectional objectifications, intersectional internalizations, intersectional blindness, and intersectional post-racism-(hetero)sexism.

### 3.1 Intersectional objectification

The research participants reported that, as AAFSMs, they were intersectionally objectified. These objectifications manifested through intersectional racialization, genderization, and sexualization. “Being an Asian queer woman is like…It’s just a lot of them are with like how like guys sexualizing, racializing and stuff…,” one participant noted (Arden, Pos. 46). Other participants agreed that these sexualizing, racializing, and genderizing processes intersected to objectify them as hypersexual, model minority, and submissive. In response to recurrent experiences of objectifications, participants also elaborated that they had to negatively adopt intersectional objectifications from others’ stereotypes and adapt to how society, institutions, and people internalized such stereotypical objectifications as normalcy.

It is important to acknowledge that objectifications are intersectional. The objectifications that the participants experienced occurred through the interactions of racialization, genderization, and heterosexualization. Research participants reported that no single objectification stood alone. For instance, with gendered objectification, race and sexuality always come into play because they are intersectionally interwoven with one another. Racialized, gendered, and sexualized objectifications intersectionally made the participants feel preferred or like a commodity because they were either sexually desirable to some White people or completely excluded from the dating pool due to their intersectional racial, gender, and sexual identities. One participant at a different Midwestern institution said, “…like you are an Asian woman and you are bisexual. Like they think it’s just like the perfect package, everything that they could dream of” (Sherron, Pos. 14). Another participant at a different institution added, “…there have been multiple occasions where um I match with guys on gender. And they say, they bring up my race cause um like I’ve never been with an Asian girl before. I’m like, I am queer, and they are like, it’s cool. It’s just like. Yeah. That’s weird. So that’s kind of like annoying” (Arden, Pos. 46). AAFSM participants always experienced intersectional objectifications, including racialization/ethnicization, genderization, and sexualization. Intersectional gendered, racial, and sexual objectifications were deemed by some White people as the perfect package and combination and all participants felt insulted and discriminated against by this “perfect package” objectification tied to their race, gender, and sexual orientation.

### 3.2 Intersectional internalizations of racism, sexism, and heterosexism

Each participant reported that they had experienced the intersectional internalizations of racism, sexism, and heterosexism. Intersectional internalizations run along a two-way street, one direction reflecting self-internalizations and the other reflecting societal internalizations. The experiences of AAFSMs revealed how self- and societal intersectional internalizations structured individuals’ lives along race, gender, and sexuality pathways.

#### 3.2.1 Internalization of racism

Internalized racism refers to self-internalization and societal, institutional, and familial internalizations of people of color. Each participant reported that they, as AAFSMs, had experienced internalized racism from both the individual level and societal level. Regarding internalizations of racism, AAFSM students viewed racial stereotypes and Whitenization as strong contributors to this process.

##### 3.2.1.1 Racial stereotypes: mismatched model minority assumptions

The participants felt they had internalized racism due to erroneous stereotypes, one of which was the model minority, which contributed to the self-internalization process of AAFSMs. This self-internalized ideology powerfully persuaded Asian Americans to act as persons they did not want to be. For instance, each participant talked about how they had to be harder on themselves because of the racial stereotypes held by their White peers. One participant elaborated,

So like yeah in my chemistry group…because of my previous experiences and people's expectations of me, I like actually try to work harder in classes…and people ask questions…I feel like they're going to expect me to know the answers. So then I kind of work harder. And I guess I didn't completely realize that until my partner pointed out like… why are you still like up? It's two a.m. like you already finished your homework. Why are you still helping them?… And I'm just like…they…they expect me to help them. And I don't know why I'm letting myself be in the situation. But…but I am… because I guess I also expect myself because of my previous experiences with people constantly saying, I should know this stuff. I'm harder on myself when I don't know something. And so, then I spend so much time on it that I feel like I expect myself to know it well enough to teach it to other people. So when they ask me a question, I expect myself to have the answer to it, which is just like kind of a frustrating cycle because I'm telling myself like, I need to stop doing this. But then I hate feeling like there's expectations, but that there are all expectations I’ve set for myself because of expectations in the past. So, then it's just this aggressive cycle where I'm trying to stop (Blake, Pos. 140).

The model minority stereotype created expectations from Asian Americans, which forced them to meet them. As Blake indicated, she did not even realize that she worked hard for simply meeting the expectation. This is a strong example of how racial minorities subconsciously internalized racial stereotypes such as the model minority myth. Participants stated additional ways in which racial stereotypes had prevented AAFSM participants from being themselves. One participant noted “Like I said, you know, I, the Asian stereotype is to be very intelligent, very smart. And I was but I tried to hide that from a lot of people so they would not see me as like, oh that She’s just that other Asian girl” (Sherron, Pos. 16). Racial stereotypes motivated Asian Americans to either hide their intelligence or be harder on themselves. Asian Americans who accepted the model minority stereotype had to be harder on themselves to meet these expectations set by others; and, those who did not accept the stereotype had to hide their intelligence to appear like others.

Oppressive behaviors toward AAFSMs were facilitated by society’s internalization of racial stereotypes such as the model minority myth. From kindergarten through college, Merritt was the only Asian in many of her classes. She described how she shaped her educational experiences to fit other people’s internalized biases and entrenched stereotypes. For instance, it was difficult for Merritt to fit in academic life because she felt a mismatch between her experiences and the racially stereotyped model minority assumptions. When asked how the model minority myth as an internalized mindset consciously and subconsciously impacted her capacity, she elaborated:

I think the whole model minority myth…it's kind of baked into a lot of people's minds, especially faculty members. So, it can go two ways, like they might think this Asian person is like really smart. Let me mentor them, let me provide more resources. Or it can go the other way where it's like this person is not asking any questions. They say we are doing great academically, but then all of a sudden you have depression and anxiety that person's dealing with and they come into the faculty’s office and then, I…I need help. And they don't help that person because they assume that they can handle it because of this model minority that has made us seem like we're robots can just do anything in academics and professional life…I think that's definitely something that's kind of just entrenched in academia in general, like this assumption that Asians and Asian Americans are inherently good at academics when that's not the case and if students are not, their self-esteem is impacted, their grades can be impacted, and their mental health can be impacted (Merritt, Pos. 65).

From a racial lens, the model minority stereotype regarding Asian Americans has been internalized into people’s mindset. In other words, in inter-group analysis (across race), the myth of the model minority becomes a normative label applied to Asian Americans. Any Asian American who did not demonstrate academic excellence remained deviant.

In addition to the inter-group analysis, the model minority myth also contributed to intra-group oppressions. For instance, one participant elaborated,

Asian Americans who internalize Model Minority myth against other Asian Americans…And I think that's the struggle with Asian Americans is because we're such a huge, diverse group that there's so many cultural differences that it's hard for us to form the same community…And I think the later on in my life, and including now I really, really hate the model minority myth. I think that it's contributing to a lot of um it's contributing to like a cycle of um oppression within the Asian American community (Merritt, Pos. 28).

Merritt indicated that within their racial group, Asian Americans were huge and diverse. Some participants who had “assimilated into White culture” did not buy into the model minority myth because they “did not want to even claim being Asian Americans” (Sherron, Pos. 81); yet others “bought into Model Minority myth” (Grace, Pos. 100). The process of internalizing the model minority stereotype was viewed as a process of internalizing racism within and across groups. AAFSMs internalized racism in multiple ways, including foreseeability on what might happen, expectations from intra- and inter-groups, and strategies for survival.

##### 3.2.1.2 Whitenization: puzzled double identity

Another theme emerged from the discussions of the participants’ dual Asian and American identities and struggles as AAFSMs. One of the factors that facilitated racism internalization was the frequent questioning by both their family culture and American culture. When asked how they internalized racism, Blake shared that her understanding on the subject was from experiences of being called “Whitewashed” by both her family members and American peers. After emigrating from the Philippines to the United States at a really young age, Blake’s understanding on internalized racism became more apparent. Living in an environment where her Asianness was questioned by her family members and her Americanness was minoritized by White peers made her feel that she “did not belong to either” (Blake, Pos. 14). Blake, as well as other AAFSMs, reported that they were often the targets of intense questioning on “Whitewashed” from both original family and institutions. “…like my cousins would call me Whitewash, even though I do not really necessarily be connected to America either” (Blake, Pos. 14). Blake continued by indicating how institutions had promoted Whitewashing as a strategy to avoid being ostracized or questioned,

…in my previous experiences, even though my university became more diverse, they still tried to push the agenda of the American dream and kind of Whitewash them rather than to just say, you're welcome here and we appreciate your culture. And it'd be nice if, like, the university systems would teach us more about other cultures even. And not just kind of dismissed them and belittle them (Blake, Pos. 189).

Without prompting, each participant revealed experiences of being called “Whitewashed” and reported how they felt insulted by the label. These feelings often compelled them to strategically accept Whitewashing for the purpose of not being ostracized, which contributed to the process of racism internalization. The participants’ understanding on internalized racism was shaped by their situational and socio-historical locations. Some interviewees recognized that their situational location had been formed to adopt Whitewashing as a strategy for acceptance. As mentioned, Merritt “tried to push away [her] Asianness and honestly kind of became um what’s the term Whitewashed” (Merritt, Pos. 12). Some interviewees revealed their struggle “over the same things as [other Filipinos] where [they]'re like Filipino Americans where [they] want to still be connected to [their] culture. But still feel connected in the American culture” (Blake, Pos. 72). It was important for them to maintain two cultures and identities. They did not want to choose one identity over the other. Socio-historically, all participants admitted that they had been perceived as “forever foreigners” through Whitenization ideology, which denied both their American and Asian identities.

Thus, inter- and intra-group expectations, entrenched racial stereotypes, previous experiences with ignorance, and self and societal pressures contributed to the racial internalization of both the participants who were AAFSMs as well as other privileged people. Participants internalized and accepted the biased racial stereotypes in order to meet their White peers’ expectations. Blake reported that she had to be harder on herself and she “hated feeling like there’s expectations, but that there are all expectations I’ve set for myself because of expectations in the past. So, then it’s just this aggressive cycle where I’m trying to stop” (Blake, Pos. 140). Meanwhile, because the participants themselves internalized racial stereotypes as the norm, White peers reinforced these stereotypes.

#### 3.2.2 Internalization of sexism

Experiences of sexism were prevalent among the AAFSM participants. Stereotypical gender roles and gender norms perpetuated oppression and contributed to the internalization of sexism. In turn, the society/institutions/White males reinforced sexism through the process of internalization.

##### 3.2.2.1 Stereotypical gender roles: Asian women as subservient females

Historically and traditionally, both Asian and American culture have been patriarchal in nature. For instance, a woman is expected to be a good wife and mother. One Filipino American female queer shared her frustration in one of the interviews, “Asian women are seen as subservient. They’re not seen as leaders” (Merritt, Pos. 75). The stereotypical gender expectation in both Asian and American patriarchal worlds set standards for Asian Americans to internalize sexism. Beliefs that “Um sexism, treating somebody differently because they present as different gender. Um uh I mean usually misogyny. Because um we live in a very patriarchal world” (Charlie, Pos. 254) were expressed many times by participants across interviews. Another participant Sherron, as one of only three women in the engineering class, demonstrated how her project had been rejected and ignored by other males, who believed Sherron should not succeed in the project.

##### 3.2.2.2 A dual gender norm for Asian American women as both home keeper and home leader

Participants reported that there was usually a dual gender norm for Asian American women. That is, they were expected to take a matriarchal role in the family and live in a patriarchal society. Many participants elaborated on the nexus between matriarchy and patriarchy. A female bisexual Filipino American graduate student from one Midwestern university narrated,

…at the same time, women fit into like traditional roles like the home keeper. And the person who cooks all the time cleans and takes care of the children. And we're expected to get married and like, just be a good wife and stuff like that. But at the same time, we have a lot of say, cause I feel like my mom's been a strong figure in my life. And she's always been kind of like the head of the household…So, it's interesting to see the duality of it. Like we have some leadership, but we have to be subservient in some sense is like we have well I mean the way that I’ve been taught… (Merritt, Pos. 30).

Misperceptions regarding matriarchy in the family and patriarchy in society have facilitated gender conceptualizations and internalizations of sexism. As Merritt continued to explain, “…that’s kind of the way I kind of started conceptualizing genders from like parents and you know how your first socialized” (Merritt Eunice, Pos. 30). Others recognized the duality of gender norms as being both socially and familially conceptualized and constructed.

Participants from various Midwestern universities discussed the unfair burden of sexism placed on AAFSMs by White Americans that facilitated the internalization process among AAFSM groups. Within society, the internalization of sexist misperceptions reinforced the gender norms that White Americans had about Asian Americans (e.g., duality).

#### 3.2.3 Internalization of heterosexism

Sexual orientation is an integral part of an individual’s identity. To authentically examine and understand how the AAFSM participants internalized heterosexism, it was important to consider parental influences and institutional influences. The following sections present the findings to indicate that parental and institutional influences facilitated the process of heterosexism internalization.

##### 3.2.3.1 Parental influences

The internalization of heterosexism was closely connected to parental influences, whereby the participants internalized heteronormativity. Each participant reported that this internalized heteronormativity often created fear. For instance, one participant narrated,

I fear, I fear about almost everyday, I'm more fearful since I'm in college, I'm away from my family. I'm more open about my sexuality… Last year, I was forced to go back home. So, I had to completely shut some part of my life away from my very traditional parents. And so, I would have to hide everything in my backpack like everything that remind that like I bought for my girlfriend and I would every day…I would fear that my parents would find them. And everything so, yeah, I it's…it’s just that fear like, now I don't have it as much now since I'm away from home. But every time I go back to my parents’ house or when my mom visits. It's like, it's there. It's still there (Dale, Pos. 18).

The most frequently mentioned reason that participants felt fear was because “[their] parents are very conservative and very traditional” (Blake, Pos. 32). Their parents’ mindsets triggered AAFSMs to believe that homosexuality was unacceptable and abnormal. Another reason our participants as AAFSMs feared coming out of the closet to their parents was because of how their parents internalized and reacted to homophobia and heteronormativity. All participants indicated that their hidden sexuality status was attributed to their parents’ internalized heterosexism; in turn, AAFSMs self-internalized non-heterosexuality as rejection, denigration, denial, and degradation. Furthermore, self-internalization and parental internalization reinforced heterosexism and heteronormativity as a societal form of oppression that (re)produced negative attitudes and policies toward AAFSMs.

##### 3.2.3.2 Institutional influences

Participants also internalized sexuality and heterosexism through religious institutions. Universities with a religious atmosphere influenced the participants’ perspectives on sexual orientation identity. For instance, LGBTQ and same-sex marriage topics were regarded as a taboo. A bisexual female Chinese American explained,

I went to …it was a Catholic university, Catholic. The Catholic church does not support homosexuality or a gay marriage. So, we were taught homosexuality is wrong. You will burn in hell, or that no good Catholic parent would keep their gay child around. They would convince me and convince my friends who are also LGBT that we were sinners, that we were going to hell, that our parents wouldn't want us if we came out. That was most definitely homophobia and heterosexism (Lyric, Pos. 140).

Our participants reported that they frequently received explicit religious messages such as “homosexuality is wrong” and “you will burn in hell” from religious institutions. These messages suggested that homosexuality and sexual minorities were shameful and abnormal. Heterosexism was very apparent on many religious campuses. Thus, our participants who were AAFSMs gradually and negatively accepted homophobia and internalized it as the norm. Even in the predominantly White public universities, heterosexism was perceived as the norm. For instance, as Merritt noted, “In general, like the term heterosexual, it’s like the assumption that everyone is straight or heterosexual. It’s the assumption that the norm is being heterosexual, that anything that falls outside of that is not natural or different and not just like. It’s like the norm” (Merritt, Pos. 87). At the Midwestern religious campuses, private and public universities where our participants studied, heterosexism was perceived as the norm.

#### 3.2.4 Intersectionality on internalization of racism, sexism, and heterosexism

Thus far, I have described how our participants as AAFSMs internalized racism, sexism, and heterosexism separately. Apparently, their experiences of racial, gendered, and sexual internalizations were not separate instances but they intersected with one another. Using the intersectionality lens allowed us to highlight how the intersections of racism, sexism, and heterosexism were internalized by the participants. Furthermore, institutional internalization formulated and perpetuated the beliefs that heteronormativity, White supremacy, and patriarchy were the established norms, impacting AAFSMs in an intersectional way. Many participants noted that it was difficult to deal with multiple oppressions that intersected with one another. This feeling was captured numerous times during interviews. It was always difficult for the group of AAFSMs because they metaphorically stood at an intersectional location, where all directions had a “dead end” sign for them.

### 3.3 Blindness and post-isms

Participants reported that they experienced intersectional blindness by White heterosexual American males. This intersectional blindness, including colorblindness, gender blindness, and sexuality blindness, has continued to silence the voices of women, has remained blind on people of color, and has denied LGBTQ. The manifestation of blindness has ranged from intentional to unintentional and from conscious to unconscious. In addition, participants reported that the racism, sexism, and heterosexism they experienced, all involved the intersection of race, gender, and sexual orientation, which we best categorized as intersectional post-racism-(hetero) sexism.

#### 3.3.1 Intersectional blindness

The participants reported that the intersectional blindness was produced by intersectional ignorance on race, gender, and sexuality. In term of race, many participants reported that their racial identity had been ignored due to other’s color blindness. For instance, one participant’s family was intentionally colorblind because her family avoided asking her, “What does it feel like to be Asian American? What does it feel like to be in an all-White family” (Sherron, Pos. 79)? When she tried to speak up for herself or make her feelings known, her White family always responded, “…I do not even really see you as being Asian, you know…you are just [Sherron] and which is fine” (Sherron, Pos. 79). Her family’s racial blindness facilitated her development and internalization of shame for being Asian American because her family’s response denied her Asianness. Sherron further explained,

You know, when you go to take I-Step in the state of Indiana, it's the standardized test. You bubbled in your race. And I had to ask like, I don't understand because I didn't know what I'm supposed to put. And that was something that was never really talked about in my house. And I was the only one that had to put something different, and it was in my you know, little mind, it’s shameful (Sherron, Pos. 48).

In this study, colorblindness intentionally removed a part of the individual’s racial identity by denying their Asianness. Colorblindness is reflective of the ignorance, devaluation, and denial AAFSMs faced. In addition to colorblindness, the participants simultaneously experienced sexuality blindness. One of the manifestations of sexuality blindness was how people viewed homosexuality as “a phase.” Each participant reported that their parents, college peers, and friends had ascribed their homosexual preference to a phase rather than admitting that it was part of that individual’s wholeness. The participants believed that their minority sexual identities were not valued by their parents, heterosexual friends, and college peers. For instance, one participant told her parents that she was bisexual. Her parents “did not understand it exactly. And they were kind of in denial. And they still kind of are, thinking oh this is a phase…Eventually she’ll meet a nice man” (Lyric, Pos. 24). A similar reflection was offered in another interview: “This is just a phase like that kind of thing” (Merritt, Pos. 38), Merritt noted that this was how her family had responded when she came out of the closet to her family. In addition to the family denial noted thus far, AAFSM students also felt insulted by their peer’s denials of their identity. One participant shared her experiences regarding coming out to peers and friends. The most frequent response she received was: “No, you are not!” (Anna, Pos. 27). These denials were best categorized as sexuality blindness because homosexuality was not acceptable or valued. Heterosexual people were not willing to accept the homosexual identity as part of the AAFSMs’ wholeness.

Gender blind is another manifestation of the blindness our participants experienced on an everyday basis. As one participant explained, “You know…[as women,] you cannot be taken…sometimes you are not even taken seriously within your profession. Um even if you could be one of the best at your craft. It’s hard…It’s extremely hard to kind of get that space” (Sherron, Pos. 95).

This intersectional blindness continuously stabilizes the dominant power while marginalizing the others. It is a way for privileged people to avoid talking about race, gender, and sexuality, which makes them uncomfortable. They are reluctant to openly discuss these topics because they fear that their social status or power might be destabilized.

#### 3.3.2 Intersectional post-racism, post-sexism, and post heterosexism

Traditional notions of racism, sexism, or heterosexism reflect how people were treated differently based on their race, gender, or sexuality. Racism, sexism, and heterosexism toward AAFSMs worsened in the post-ism era. This post-ism era includes post-racism, post- sexism, and post-heterosexism, which are far more complex and intersectional. Individuals’ race, gender, or sexual identities inevitably interplay with other aspects of their identity, generating genderized-sexualized-racism, racialized-genderized-heterosexism, or sexualized-racialized- sexism.

For instance, one participant, who is a bisexual female Chinese American, noted that her White mother did not understand race-related feminism,

like she considers herself like a feminist. And ah but in the beginning, when I started talking to her about like race, she didn't understand that like me being a minority is somehow like different and like different aspects than just being like a woman because she's a White woman. And honestly, I think they work through the world much differently than we do like based on like race like gender discrimination. Like I'm very aware of that. But they also have certain privileges up the belt. And like, I have to deal with a lot of stuff that she's like never even like thought about. Oh, so that was like hard because like she kept trying to be like oh like in a like totally like understand where you're coming from. Like it's hard being a member of like group that's like oppressed. And I'm like, yeah but I'm not just oppressed by one. It's like multiple and like it affects me like differently from you (Arrow, Pos. 50).

Traditional feminists did not take race into full consideration because they mainly gave emphasis to White women. The post-feminist became more intersectional because its manifestations were racialized feminist or sexualized feminist. Traditional feminists primarily objected to the oppression of White women while neglecting other oppressed groups, which were oppressed not only by sexism but also by racialized sexism and sexualized sexism.

Similarly, traditional racism, as understood by participants, mainly emphasized race. By contrast, post-racism placed AAFSMs at the intersection of gendered racism and sexualized racism. Numerous experiences of racism were noted in the interviews, and many involved gender and sexuality. For instance, as mentioned in the previous section, a female queer Filipino American narrated, “Asian men are like, not sexualized, right? Like they are…there is no sex appeal to Asian men. Asian women are hypersexualized” (Lennon, Pos. 40). The participants believed this tokenization applied not only to racism, but also extended to include genderized racism and sexualized racism.

Heterosexism is an entrenched oppression that discriminates against sexual minorities. Yet the experiences of heterosexism by AAFSMs were distinctive and complex. To examine and understand how heterosexism is experienced, it is important to take into consideration post-heterosexism such as genderized heterosexism and racialized heterosexism. Post-heterosexism as genderized heterosexism was captured in an interview with a bisexual female Chinese American who shared her feelings of frustration regarding her heterosexual White male college peer’s negative attitude toward LGBTQ:

I mentioned it like…like men are okay with like women being like bisexual, but not men. So, he definitely was…homophobic towards like men being gay. And like that was really frustrating to me because I'm like…there's no different than me, being with a woman and like a guy being like a man. And you're just like, oh it's like gross. And like, that was I don't know. That was like so frustrating to me (Arrow, Pos. 86).

All participants reported that heterosexism was genderized because female LGBTQs were more acceptable in comparison to male gays. Another racialized heterosexism was brought to light in an interview with a female queer Filipino American:

Because I think when you're an Asian lesbian, there's a lot of fetishization. And um it's this weird mix of guys in particular think that you're ugly. But then some of them think that you're really attractive. And it's like, where exactly do I fall? Right? (Merritt, Pos. 12).

Similar feelings were expressed by many participants who believed that their unstable status was not merely based on racism, sexism, or heterosexism. Instead, they experienced intersectional post-racism, -sexism, and -heterosexism. Its manifestation could be either racialized-genderized-heterosexism, racialized-sexualized-sexism, or sexualized-genderized-racism.

## 4 Conclusions and discussion

The study examined the complex interplay of racism, sexism, and heterosexism as experienced by Asian American and Pacific Islander (AAPI) female students with multiple marginalized identities.

### 4.1 Puzzled double identity

Participants expressed significant struggle and puzzle with their dual identities as Asian and American. The theme of puzzled double identity highlights the internal conflict experienced by Asian American female students (AAFSMs) who navigate between their cultural heritage and their American identity. The frequent questioning of their “Asianness” by their family and “Americanness” by their peers led to their feelings of not belonging to either culture. Participants were labeled as Whitewashed by both their families and peers, a term that signifies a perceived loss of cultural authenticity due to assimilation into mainstream American culture. This conflict often led to a sense of not fully belonging to either cultural sphere, contributing to internalized racism and identity confusion. This dynamic fostered a form of internalized racism, where participants internalize racist stereotypes and ideologies from a dominant White society. Such internalization leads to self-doubt, self-disgust, and disrespect for one’s own racial group and makes participants feel inferior and psychologically struggled and puzzled with their dual identity.

The concept of Whitewashed reflects the pressures on AAFSMs to conform to dominant cultural norms while simultaneously being judged by their own cultural communities for not meeting traditional expectations. This dual pressure can lead to significant psychological stress and identity struggles. Internalized racism among minority groups often stems from such conflicting expectations and stereotypes imposed by both their ethnic communities and the broader society.

### 4.2 Internalization of sexism

The participants in the study experienced the internalization of sexism through traditional gender roles and patriarchal expectations from both Asian and American cultures. Gender norms placed Asian American women in a contradictory position, expecting them to be subservient at home while simultaneously being pressured to take leadership roles in a patriarchal society. Such dual internalization of sexism created conflicting gender norms for AAFSMs. This dual expectation perpetuated the internalization of sexism, with women often feeling constrained by societal expectations that conflicted with their personal experiences and ambitions. This internalized sexism is reflective of broader patriarchal systems that dictate gender roles and expectations. Traditional gender roles often reinforce stereotypes and limit their opportunities. The participants’ experiences reflect a struggle between fulfilling these expectations and asserting their individual identities and ambitions.

### 4.3 Internalization of heterosexism

The internalization of heterosexism among AAFSMs was significantly influenced by their conservative family backgrounds and institutional environments that stigmatized non-heteronormative identities. Participants reported fear and concealment of their sexual orientations due to the pressure from conservative values and religious institutions that deemed non-heteronormative identities as unacceptable. This internalization reinforced a negative self-perception and the denial of their sexual orientation, manifesting in self-censorship and a disconnection from their authentic selves. This phenomenon aligns with the concept of heteronormativity, where non-heterosexual identities are marginalized and stigmatized.

### 4.4 Intersectional blindness

Participants experienced various forms of intersectional blindness—colorblindness, gender blindness, and sexuality blindness—where their racial, gender, and sexual identities were ignored or invalidated by others. This blindness, both intentional and unintentional, perpetuated dominant power structures while marginalizing their voices (Crenshaw, 1991). The concept of post-isms reflects how traditional notions of racism, sexism, and heterosexism have evolved into more complex and intersecting forms, such as genderized-sexualized racism and racialized-gendered heterosexism.

The concept of intersectional blindness highlights how dominant groups often ignore or invalidate the intersecting identities of marginalized individuals. This blindness perpetuates systemic inequalities and makes it difficult for marginalized individuals to have their experiences and identities acknowledged. Intersectional blindness reflects the refusal or inability of privileged groups to recognize the complexity of intersecting identities. Colorblindness and other forms of blindness maintain the status quo of the dominant groups by disregarding the nuanced experiences of marginalized groups (Crenshaw, 1991; McCall, 2005).

Although, the findings cannot be generalized to the experiences of all AAFSM college students, this qualitative study does contribute to the body of literature by suggesting how educators and policymakers can provide an intersectional inclusive educational environment for AAFSM students attending Midwestern universities. The suggestions and implications include: (1) de-internalizing the intersectional internalizations, (2) implementing intersectionality, and (3) increasing intersectional connections, visibilities, and representations.

### 4.5 Intersectional-internalization, de-intersectional-internalization, and re-intersectional- internalization

In the context of racial, gendered, and sexual stratifications, intersectional internalization is a process whereby our participants negatively accept devaluation, marginalization, and low socio-historical and socio-cultural status regarding their race, gender, and sexuality identities. Meanwhile, their acceptance is facilitated and reinforced by the dominant ideologies of White supremacy, patriarchy, and heteronormativity. The intersectional internalizations on race, gender, and sexuality were exemplified by denying their Asianness, degrading their homosexuality, devaluing their womanhood, and strategically accepting the White heterosexual male as superior to not feel ostracized. Both self-internalization and social and institutional internalizations burdened our participants through intersectional oppressions, exclusions, and discrimination. The findings supported our contention that participants experienced intersectional racism, sexism, and heterosexism by internalizing racial stereotypes, Whitenization, gender norms, stereotypical gender roles, and parental, social, and institutional beliefs. Our research findings also contributed to the literature by showing that the aforementioned factors served as internalizing function for both our participants and White/society/institutions insofar as they internalized stereotypical norms.

As a result, these internalized norms in the mindsets of both the marginalized and the dominant White/society/institution (re)produced intersectional inequalities and reinforced heterosexual White males’ social status. In other words, the findings showed that participants internalized the intersectional social hatred and then, in turn, produced intersectional internal phobia, which oppressed them in an intersectional way. Specifically, both heterosexual and homosexual individuals internalized sexual related social hatreds, such as homophobia, biphobia, and transphobia, and then formed internal phobia. The sexual related phobia internalized by heterosexual people reinforced the social norms and heteronormativity. At the same time, the phobias internalized by sexual minority individuals caused self-oppression. Gender-related and race-related internalizations applied as well.

From an intersectional perspective, multiple societal stereotypes and the norms of the dominant culture can facilitate a marginalized individual to internalize intersectional phobias such as homophobia, biphobia, transphobia, xenophobia, racism, sexism, heterosexism, etc. Therefore, we suggest that marginalized minorities should de-internalize these intersectional norms. Privileged people should de-internalize their entrenched biases on race, gender, and sexual orientation, which tend to result in intersectional inequalities and discrimination toward our participants. Universities should motivate students to adopt a “blank slate” when learning about new cultures. This “blank slate” attitude can de-internalize societal stereotypes and biases embedded in people’s mindsets and re-internalize new understandings.

### 4.6 The implementation of intersectionality

Overall, the study highlights the complexities faced by AAFSMs due to the intersection of their racial, gender, and sexual identities. To create a more equitable and supportive academic and social environment for AAFSMs and similar marginalized groups, educational institutions can adopt inclusive practices, provide targeted support, and foster environments that challenge stereotypes and address the internalized effects of discrimination.

Our findings were consistent with assumptions that our participants’ race, gender, and sexual orientation shaped their lived and educational experiences intersectionally. They reported that their lived and educational experiences were unique. Racially, they were sometimes lumped with White people because they had lighter skin color. Nevertheless, they were considered as “perpetual foreigners” (Tuan, 1998). Sometimes, they were grouped with Asians due to their visibility of Asianness. Gender wise, they were grouped as weak, submissive, and “not useful” (Lyric, Pos. 82). Sexually, our participants were grouped as sick. Our research showed that the grouping of our AAFSM participants into multiple derogatory categories was a form of oppression. Our findings also indicated that no one fit into a certain group perfectly. Grouping produces boundaries and limitations. Therefore, the implementation of intersectionality at the institutional level is extremely important. Intersectionality operates as a critical ideology and theory to deconstruct groupings and eliminate generalizations about an entire group of people. On campuses, the ideology of intersectionality should be implemented in student organizations such as Asian American students’ organization, women’s club, LGBTQ organization, etc. Each participant noted that the focus on one master marginalized identity (i.e., race, gender, or sexuality) in student organizations was a way to ignore individuals’ invisible identities, considering only the master identity limited people’s understanding, which (re)produced intersectional inequalities and oppression. As the participants reported that they did not neatly fit into any of these overarching programs because of their intersectional identities. For instance, Bourgeois (2014) indicated that “over 600 Women’s Study programs have been established at colleges and universities…[to] focusing on women’s varied and unique experiences” (p. 6). Asian Americans who are female and sexual minorities do not fit into most of the overarching programs. Greene (1997) demonstrated that “the process of acknowledging an LGB[TQ] orientation (i.e., ‘coming out’) may translate into greater potential for losses because of the number of communities of which LGB[TQ] People of Color may be a part” (also cited in Miville and Ferguson, 2006, p. 95). In addition, to solve the problems that marginalized students faced on a daily basis, the above-mentioned programs used a “one-at-a-time approach” (Collins and Bilge, 2018, p. 3) that emphasized a master marginalized identity. The consistency between our findings and arguments mentioned previously confirmed that intersectionality needs to be implemented into student organizations in Midwestern universities. Specifically, in Asian American student organizations, visible (e.g., gender) and invisible identities (e.g., sexuality) should be welcomed and appreciated. Employing and implementing intersectionality in each student organization on campus is extremely important because it could actively make invisible identities more visible. Intersectionality, we believe, is a breakthrough idea that serves to deconstruct the dominance of a master identity on people. It inspires people to view individuals in a more holistic way, rather than using a limited and stereotypical lens.

In addition to student organizations, we also suggest introducing intersectionality in class discussions, conferences, and seminars on campuses. Many privileged people felt exhausted and were reluctant to talk about race, gender, sexuality, etc. because they had been listening to the minority experience for a long time. However, they had not listened carefully to marginalized people. On-campus academic activities that promote intersectionality can provide opportunities for privileged people to listen again to others’ experiences, this time with careful attention, because the post-racial, -gendered, and -sexual era is different from the traditional one. Intersectionality reminds privileged people to carefully re-listen to the voices of multiple marginalized individuals whose experiences are intersectional, complex, and different.

### 4.7 Intersectional connections, visibilities, and representations

The consistency between our findings and those of prior literature confirmed that much remains to be done to make university campus environments more inclusive for students with multiple marginalized identities. University administrators should increase visibility and representation of both tangible (e.g., race, gender, etc.) and intangible (e.g., sexuality) identities on campus. Depending on the situation and context, the participants felt that they needed to strategically put certain identities into the foreground or background to survive in classes and on campus. Many referenced the lack of intersectional visibility and representation on campus, which disconnected participants from others. Connections and disconnections emerged simultaneously when an individual’s visible identities were perceived while the invisible identities were ignored. Therefore, increasing intersectional visibility and representation regarding both visible and invisible identities can provide a wide range of connections leading to a more inclusive environment.

First and foremost, we suggest that universities, especially predominantly White universities, should increase the population of people with multiple marginalized identities, including professors, staff, students, and administrators. Many participants reported that they felt disconnected, ignored, and excluded because “there’s not enough of us on campus” (Charlie, Pos. 250). Another participant shared a similar experience, “None of my professors have ever explicitly told me their sexual orientation. So, I am actually not sure if any of them were LGBTQ or not…I’ve never had an Asian professor…Everyone else has been White” (Lyric, Pos. 136). Our findings suggest that increasing visibility or representation of professors with multiple marginalized identities could make students feel safer. One participant asserted, “I can tell you that for sure, if I had an LGBTQ professor, I would immediately feel more comfortable around them” (Lyric, Pos. 134). In addition, professors, educators, and administrators should actively and intentionally deliver positive messages regarding intersectionality to make invisible identities more visible to both marginalized and privileged students. In environments that did not explicitly embrace the ideas of intersectionality, students were reluctant to disclose their identities, which oppressed and disconnected them. One participant elaborated,

There’ve been a couple of times where doing essays in class or something, the topic has come up and I have to find a way to either just tell them that I’m not straight or hide it, because I’m not sure if the professor will end up discriminating against me or not (Lyric, Pos. 136).

Furthermore, I suggest that each campus student organizations should implement intersectionality to increase awareness about invisible identities, thereby providing greater opportunities to forge connections. For instance, participants offered numerous examples of situations in which they felt disconnected in the LGBTQ student organization, Asian American student organization, or the women’s club. Due to the lack of intersectional visibility and representation in these groups and their focus on one marginalized identity, the AAFSM students related that although they felt connected to the master identity, they felt disconnected from other parts of themselves. One participant shared her feelings regarding the LGBTQ student organization, “There is like places I can relate to as because I’m a lesbian…But I as someone who is in multiple [race, gender, sexuality], it’s like…I cannot relate to everything” (Dale, Pos. 10). Therefore, increasing visibilities and representations in student organizations is one way to connect individuals and provide an intersectional inclusive campus for students with multiple marginalized identities.

In short, our findings indicated that the reason the AAFSM participants felt disconnected from their Asianness, womenness, and LGBTQness was due to the scarcity of intersectional representation and visibility. Therefore, increasing intersectional visibility and representation could reconnect AAFSMs with their Asianness, womanhood, and LGBTQness.

### 4.8 Intersectionalism

Our findings extended the work—increased intersectional connections, visibility, and representation—by suggesting that the implementation of affirmative action initiatives that support campus diversity did not effectively eliminate intersectional inequalities. As I elaborated previously, our research participants reported that they experienced intersectional exclusions directly related to intersectionalism. This intersectionalism included intersectional objectification, intersectional internalization, intersectional blindness, and intersectional post-racism-(hetero) sexism. Therefore, we suggest three steps that Midwestern universities should implement to eliminate intersectional exclusions. These three steps are: (a) intersectional racial-gendered-sexual awareness, (b) intersectional racial-gendered-sexual values, and (c) intersectional racial-gendered-sexual emancipations. Implementation of these steps could be best categorized as omniculturalism, creating an intersectional inclusive campus environment.

The first step is to raise intersectional racial-gendered-sexual awareness. Specifically, educators, educational administrators, and universities should foster intersectionality through academic activities, teaching, curriculum content, etc. to raise people’s awareness about race, gender, and sexual orientation via an intersectional lens. This first step is designed to de-internalize entrenched biases, stereotypes, and prejudices that privileged people, institutions, and society held regarding individuals with multiple marginalized identities.

The second step that I suggest, intersectional racial-gendered-sexual values, aims to give equal values to Asianness, womeness, and LGBTQness. These findings are consistent with the notion that devaluation on Asianness, womenness, and LGBTQness was associated with power relations. These power relations prioritize heteronormativity, White supremacy, and masculinity. The second step aims to deconstruct the power relations socio-historically constructed by privileged people, society, and institutions. Further, universities need to give intersectional equal values to racial, gender, and sexuality minorities to eliminate intersectional inequalities produced by intersectional power relations.

The last step aims, ideally and eventually, to emancipate the participants who are AAFSMs. The emancipation means to prosper AAFSM students by valuing their cultures equally. In addition, emancipation also means that universities should provide equal and equitable educational opportunities and resources to this population.

Implementation of intersectionalism in universities, especially Midwestern predominantly White universities, can foster an intersectional inclusive campus environment for students with multiple intersectional identities.

## 5 Limitations

A limitation of this study is its reliance on *intra-categorical complexity*, which, while necessary for managing the scope of the research, inherently constrains the analysis by focusing on only one dimension within each analytical category—race (Asian American), gender (women), and sexuality (sexual minority). This approach, while useful for simplifying the complexity of intersectionality, inevitably overlooks the nuanced experiences of individuals who may occupy multiple or fluid positions within these categories, such as those identifying as non-binary or belonging to specific ethnic subgroups within the Asian American community. As a result, the study may not fully capture the diversity of experiences within the broader categories selected, potentially limiting the generalizability and depth of the findings.

The second limitation of this study lies in its reliance on in-depth interviews and open-ended questions as the primary data collection methods. While these approaches were essential for capturing detailed and holistic narratives of AAFSM college students’ experiences with race, gender, and sexual orientation, they may also introduce potential biases and limitations. For instance, the depth and quality of the data collected could be influenced by the participants’ ability and willingness to articulate their experiences, which may vary widely. Additionally, the modifications made to the interview protocol based on the pilot study, while aimed at enhancing clarity and engagement, may not have fully addressed all possible sources of misunderstanding or discomfort during the interviews. The use of follow-up interviews to clarify responses is a strength, but it also highlights the challenge of ensuring comprehensive and consistent data across all participants. Finally, while efforts were made to protect the confidentiality of the participants, the small sample size and the specificity of the group may still pose a risk of identification, potentially impacting the openness of the participants’ responses.

## 6 Significance and future research

Intersectionality provides a framework for understanding how multiple forms of oppression intersect and affect individuals in complex ways. The term intersectionality describes how various forms of discrimination, such as race and gender, overlap and create compounded experiences of marginalization (Crenshaw, 1991).

The study highlights how racism, sexism, and heterosexism intersect, leading to intertwined experiences of oppression. Participants faced a convergence of multiple forms of discrimination, making it difficult to navigate their identities and experiences. The intersectional lens revealed how these overlapping oppressions were not isolated but interconnected, creating a unique and complex pattern of marginalization for AAFSM female students.

The concept of intersectionality appears with great frequency in many scholarly fields in social science such as educational studies. This study on intersectionality drew attention toward an under-represented AAFSM college student group and stressed that their voices should be heard. The study employed the lens of intersectionality to explore how issues of race, gender, and sexual orientation had intersectionally impacted the AAFSM college student participants as they navigated through education. The results provided crucial information for stakeholders (e.g., school policymakers) to develop interventions designed to create an intersectional-inclusive educational environment. The study added a new field of scholarship dedicated to the study of AAFSMs, and added further knowledge to the study of what is known as intersectionality scholarship. The meaningful contributions include intersectional objectifications, intersectional internalizations, intersectional blindness, and intersectional post-racism-(hetero) sexism.

For future studies, emerging scholars, student researchers, and educators could employ McCall’s (2005) other two methodological approaches—anti-categorical complexity and inter-categorical complexity—to explore the intersectionality studies. In terms of interviews in intersectionality studies, we directly referenced the concept of intersectionality conducting interviews. Some intersectionality researchers (e.g., Windsong, 2016) argued that interview questions needed to incorporate intersectionality directly and precisely. For instance, the questions in this study were constructed as “how do you describe your educational experiences with regard to race and sexuality as a woman?” Alternately, Windsong (2016) suggested that researchers “craft interview questions that specifically incorporated intersectionality” (p. 141). Windsong (2016) argued that when interview questions regarding race and gender being were asked separately, participants did not share their perspectives from an intersectionality view. However, some researchers argued that directly pointing out the theme intersectionality in the interviews could impact the authenticity of the study if the theme of intersectionality did not emerge naturally. Therefore, future research should be conducted using two different interview designs. Most importantly, intersectionality aims to explore how an individual’s specific location shapes or influences them in specific ways, instead of merely including an endless list of multiple identities. The list of identities is endless because it will never be completed. Therefore, Davis (2014) argued that “such a list does not do much work and may, ironically, even end up becoming an excuse for not doing the necessary analysis of situating one’s self” (p. 20). Therefore, last but not the least, suggestions on intersectionality study is to explore how intersectional identities interact with interlocking systems of oppressions and exclusions.

## Data Availability

The raw data supporting the conclusions of this article will be made available by the authors, without undue reservation.
